# SUMO1-regulated DBC1 promotes p53-dependent stress-induced apoptosis of lens epithelial cells

**DOI:** 10.18632/aging.205001

**Published:** 2023-09-07

**Authors:** Yan Wang, Jing-Miao Wang, Yuan Xiao, Xue-Bin Hu, Shu-Yu Zheng, Jia-Ling Fu, Lan Zhang, Yu-Wen Gan, Xing-Miao Liang, David Wan-Cheng Li

**Affiliations:** 1 State Key Laboratory of Ophthalmology, Zhongshan Ophthalmic Center, Sun Yat-Sen University, Guangzhou, Guangdong 510060, China

**Keywords:** lens, cataract, apoptosis, DBC1, p53, oxidative stress

## Abstract

Deleted in breast cancer 1 (DBC1) was initially identified from a homozygously deleted region in human chromosome 8p21. It has been well established that DBC1 plays a dual role during cancer development. Depending on the physiological context, it can promote or inhibit tumorigenesis. Whether it plays a role in lens pathogenesis remains elusive. In the present study, we demonstrated that DBC1 is highly expressed in lens epithelial cells from different vertebrates and in retina pigment epithelial cells as well. Moreover, DBC1 is SUMOylated through SUMO1 conjugation at K591 residue in human and mouse lens epithelial cells. The SUMOylated DBC1 is localized in the nucleus and plays an essential role in promoting stress-induced apoptosis. Silence of DBC1 attenuates oxidative stress-induced apoptosis. In contrast, overexpression of DBC1 enhances oxidative stress-induced apoptosis, and this process depends on p53. Mechanistically, DBC1 interacts with p53 to regulate its phosphorylation status at multiple sites and the SUMOylation of DBC1 enhances its interaction with p53. Together, our results identify that DBC1 is an important regulator mediating stress-induced apoptosis in lens, and thus participates in control of lens cataractogenesis.

## INTRODUCTION

Apoptosis was first proposed by a group of British scientists, referring to the special type of death where membrane blebbing, nuclear condensation and DNA degradation were followed by endocytosis by the neighboring cells [1–2]. Apoptosis plays an important role in ocular pathogenesis [3–6]. In the ocular lens, we have previously demonstrated that the induced apoptosis by various stress factors appear to be a common cellular basis for non-congenital cataractogenesis [7–9]. These earlier studies were subsequently confirmed from *in vivo* studies by different groups including our own work [10–15]. Moreover, interruption of normal lens physiology by overexpressing exogenous genes or silence of endogenous genes all induces apoptosis followed by lens pathology [16–27].

Apoptosis is regulated by various positive and negative regulators [28–31]. One of the master regulators for apoptosis is p53, a tumor suppressor [32–35]. Silence of p53 function leads to inactivation of apoptosis in many types of tumor cells as well as non-tumor cells [32]. It is well established that p53 can regulate apoptosis through different mechanisms. First, as a transcription factor, p53 regulates several dozens of apoptosis-related genes [35]. In this regard, we have previously shown that p53 can regulate Bak, a major pro-apoptotic gene, to mediate apoptosis and lens differentiation [36]. In addition, p53 can activate Bax in the mitochondria to interact with Bcl-2 and Bcl-XL [37, 38].

DBC1 was initially identified in a frequent homozygous deletion region in breast cancers and presumed to be a tumor suppressor [39]. Later studies showed that DBC1 plays a dual role in tumorigenesis, either promoting or inhibiting cancer development [40–43]. Recently, numerous laboratories have shown that DBC1 plays multiple roles in physiology, such as being a coactivator of some nuclear receptors [44–46]; acting as an endogenous inhibitor of SIRT1 [47–49], HDAC3 [50] and SUV39H1 [51] that regulates these components of the epigenetic modifiers. Whether DBC1 plays a role in the ocular lens remains elusive.

SUMOylation is an important post-translational modification where small ubiquitin-like modifiers (SUMOs) are conjugated with substrate proteins at a conserved lysine residue [52, 53]. The covalent conjugation of SUMO to its substrates involves a three-step enzymatic cascade consisting of E1 activating enzyme (SAE1/UBA2) [54], E2 conjugating enzyme (Ubc9) [55] and E3 ligases (PIASs) [56, 57]. So far, five SUMO isoforms have been identified in human. SUMO1-SUMO3 are ubiquitously expressed while SUMO4 and SUMO5 are only expressed in specific tissues [58, 59]. The sequence identity of SUMO1 and SUMO2/3 is less than 50% [60]. In contrast, SUMO2 and SUMO3 are nearly identical (about 97% in humans) and cannot be distinguished by antibodies. For this reason, they are often referred to as SUMO2/3 [61]. SUMOylation is reversed by SUMO specific proteases (SENPs) that cleave SUMO from the substrate [62]. SUMOylation participates in the control of various cellular processes, including DNA replication [63], gene transcription, cell cycle regulation [64], DNA damage repair [65], chromatin organization [66], and signal transduction [53]. In the ocular lens, we have demonstrated that SUMOylation of several transcription factors including Pax6 and Sp1 is involved in control of lens differentiation [67, 68]. More recently, we found that during cataractogenesis, Pax6 SUMOylation is much enhanced in cataract patients. Moreover, SUMOylation ligases UBA2, Ubc9, PIAS1, as well as the de-SUMOylation enzyme SENP2/6 are upregulated in lens epithelia of the 50–70 year old patient groups, enhancing the SUMOylation patterns of various target proteins [69]. One of the target proteins is the tumor suppressor, p53. We demonstrated that the E3 ligase PIAS1 regulates p53 SUMOylation to promote oxidative stress-induced apoptosis of lens epithelial cells [70]. On the other hand, de-SUMOylated p53 is capable of recruiting heterochromatin to the promoters of the downstream target genes and thus suppresses stress-induced apoptosis of retina pigment epithelial cells, preventing occurrence of age-related macular degeneration in retina [71].

In the present study, we demonstrated for the first time that DBC1 is highly expressed in human and mouse lens epithelial cells. DBC1 is localized in the nucleus where it becomes SUMOylated through SUMO1 conjugation. Functionally, it can promote stress-induced apoptosis, and thus participating the control of cataractogenesis [7–9]. Mechanistically, DBC1 can interact with p53 to modulate the phosphorylation status at multiple sites, and its SUMOylation enhances the interaction with p53. Together, our results identify that DBC1 is an important regulator mediating stress-induced apoptosis in lens. Through promotion of stress-induced apoptosis, DBC1 is implicated in control of formation of cataract, a leading ocular disease that causes global blindness [72].

## RESULTS

### DBC1 is highly expressed in different ocular cell lines

**Figure 1:**
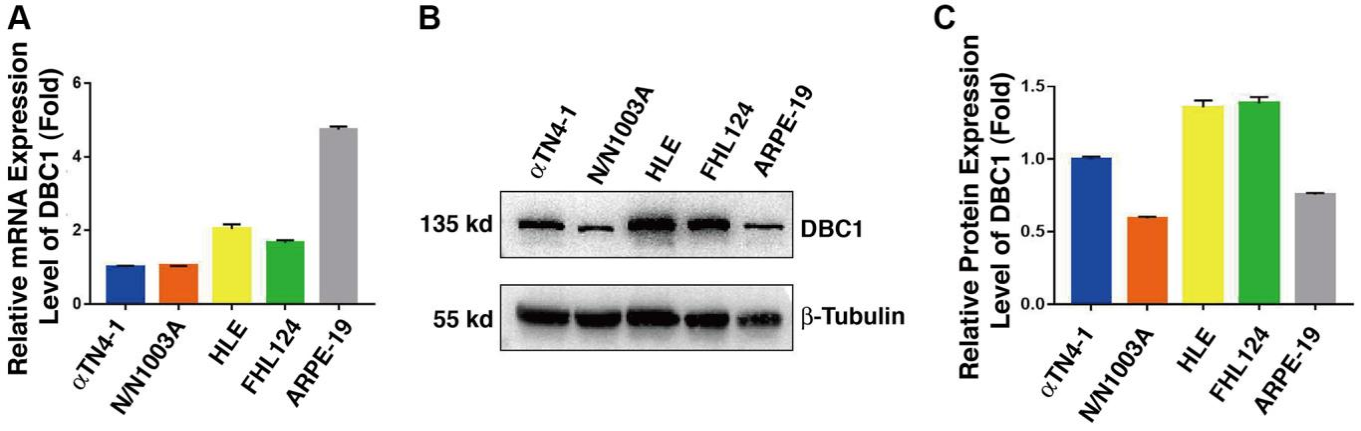
**DBC1 expression patterns in different ocular cell lines.** (**A**) Quantitative RT-PCR analysis showed mRNA levels of DBC1 in four different lens epithelial cell lines (αTN4-1, N/N1003A, HLE, FHL124) and one retinal pigment epithelial cell line (ARPE-19). Ct values were normalized by β-actin for each sample. The primers of DBC1 and β-actin were designed from mouse, rabbit and human species, the sequence details can refer to Supplementary Table 1. (**B**) Western blot analysis of DBC1 protein level in these ocular cell lines. β-tubulin served as the loading control. (**C**) Quantification of the Western blot results in panel (**B**).

In order to analyze DBC1 function in the ocular lens, we first analyzed the expression patterns of DBC1 in lens cell lines derived from different vertebrates and also retina pigment epithelial cells. qRT-PCR and western blot analysis were used to examine the mRNA and protein levels of DBC1 in 4 lens cell lines: αTN4-1 (mouse lens epithelial cells), N/N1003A (rabbit lens epithelial cells), HLE (human lens epithelial cells containing a SV40 large T antigen) and FHL124 (human embryonic lens epithelial cells), and a human retinal pigment epithelial cell line: ARPE-19. As shown in Figure 1A, the mRNA level of DBC1 was highest in ARPE-19, followed by HLE and FHL124, and lower in αTN4-1 and N/N1003A. In contrast, the protein expression of DBC1 in human (HLE and FHL124) and mouse (αTN4-1) lens epithelial cells displayed the highest levels (Figure 1B, 1C), suggesting that DBC1 may play a crucial role in these cells.

### DBC1 is localized in the nuclei with SUMO1-conjugation in different ocular cell lines

**Figure 2:**
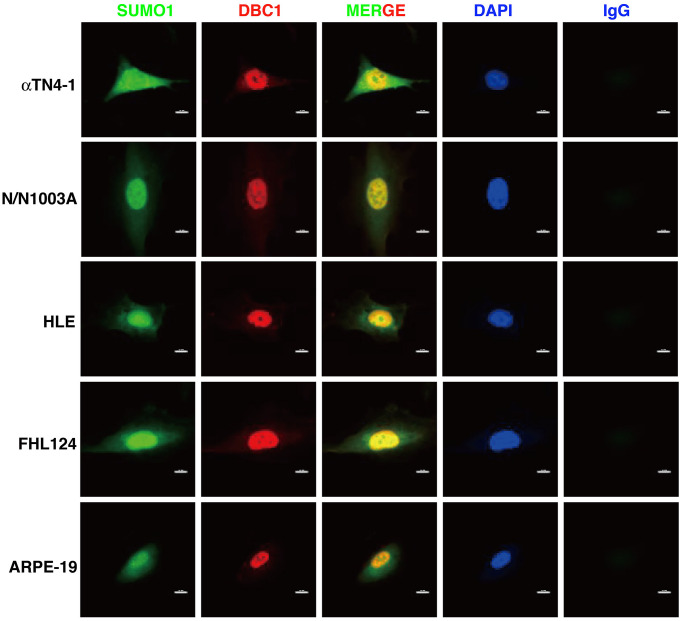
**Immunofluorescence analysis of DBC1 in the five ocular cell lines.** Note that DBC1 (red) was located in the nuclei (DAPI staining, blue), while SUMO1 (green) was located in the nuclei and cytoplasm, co-localized with DBC1 in the nuclei. IgG served as a negative control. Scale bar, 12 μm.

Next, we used immunofluorescence to determine the subcellular localization of DBC1 in different cell lines described above. As shown in Figure 2, DBC1 is clearly localized in the nuclei, which is consistent with previous studies in non-ocular cell lines [73]. Since our previous studies have revealed that specificity protein 1 (Sp1), a major transcription factor that controls expression of lens-specific genes such as β-crystallins, was positively regulated by SUMO1 but negatively regulated by SUMO2/3 [68], we speculated that DBC1 may be medullated by SUMO1 conjugation in the lens epithelial cells. As shown in Figure 2, indeed, we detected a distinct nuclear colocalization between DBC1 and SUMO1 in 4 lens epithelial cell lines as well as in retina RPE cells, suggesting that DBC1 is SUMOylated by SUMO1 conjugation in these ocular cell lines.

### DBC1 is SUMOylated through conjugation with SUMO1 but not SUMO2/3 in mouse lens epithelial cells

**Figure 3:**
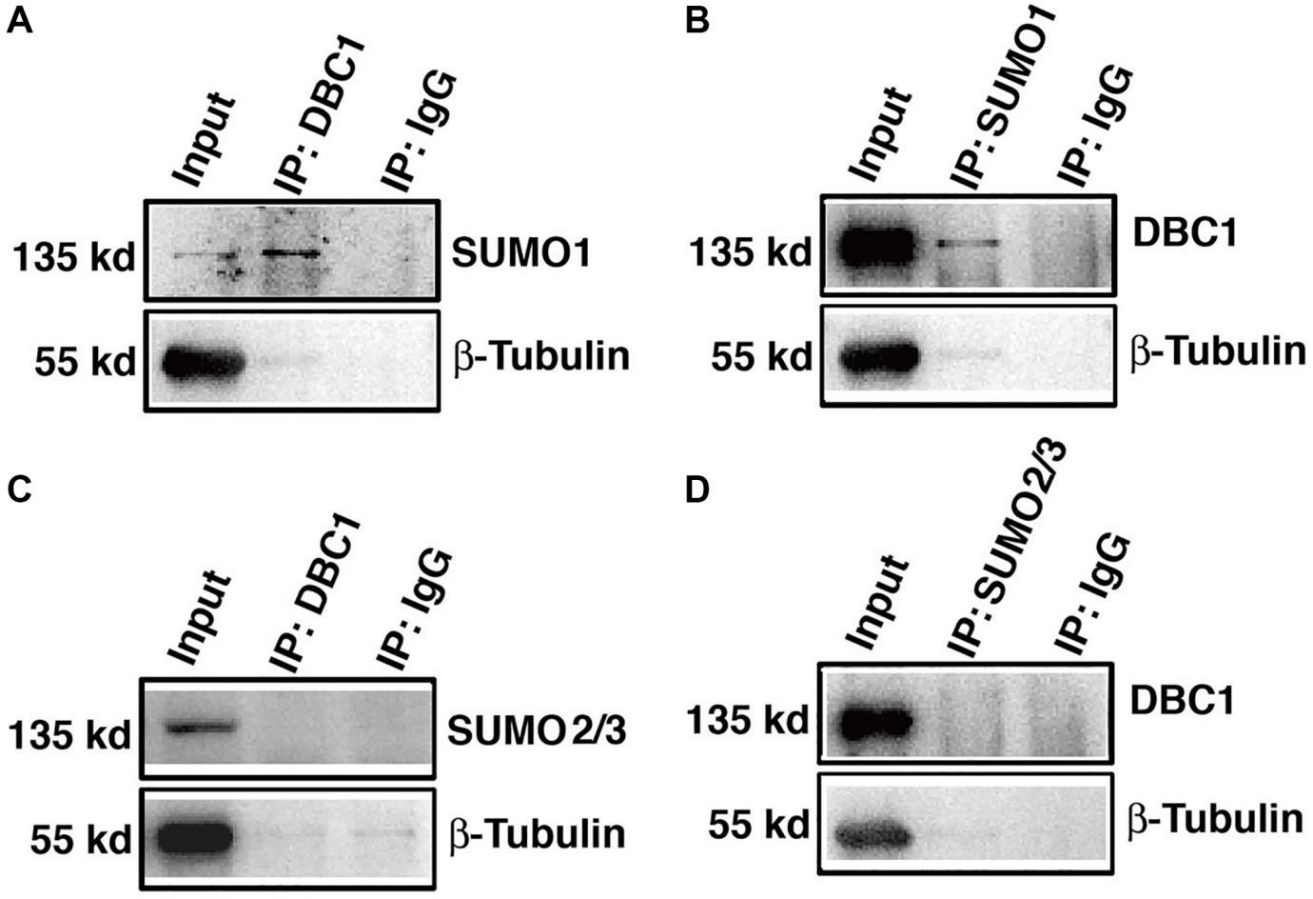
**DBC1 is modified by SUMO1 rather than by SUMO2/3 in mouse lens.** (**A**, **B**) The cell lysates of wildtype mouse lens epithelium were immunoprecipitated (IP) with anti-DBC1 (A) or anti-SUMO1 (B) antibody, followed by immunoblotting with anti-SUMO1 (**A**) or anti-DBC1 (**B**) antibody. (**C**, **D**) The cell lysates of wildtype mouse lens epithelium were immunoprecipitated (IP) with anti-DBC1 (**C**) or anti-SUMO2/3 (**D**) antibody, followed by immunoblotting with anti-SUMO2/3 (**C**) or anti-DBC1 (**D**) antibody.

To confirm that DBC1 is indeed SUMOylated by SUMO1 conjugation, we dissected lens epithelium tissue of C57BL/6J mice and conducted co-immunoprecipitation (Co-IP) assays with anti-DBC1 and anti-SUMO1 or SUMO2/3 antibodies, respectively. As shown in Figure 3A, 3B, we can detect an obvious interaction between DBC1 and SUMO1 of mouse lens epithelium and vice versa. However, the Co-IP analysis between DBC1 and SUMO2/3 revealed absence of the interaction between them (Figure 3C, 3D). Thus, DBC1 is SUMOylated through conjugation with SUMO1 but not SUMO2/3 in mouse lens epithelial cells.

### The K591R mutation prevents DBC1 SUMOylation

**Figure 4:**
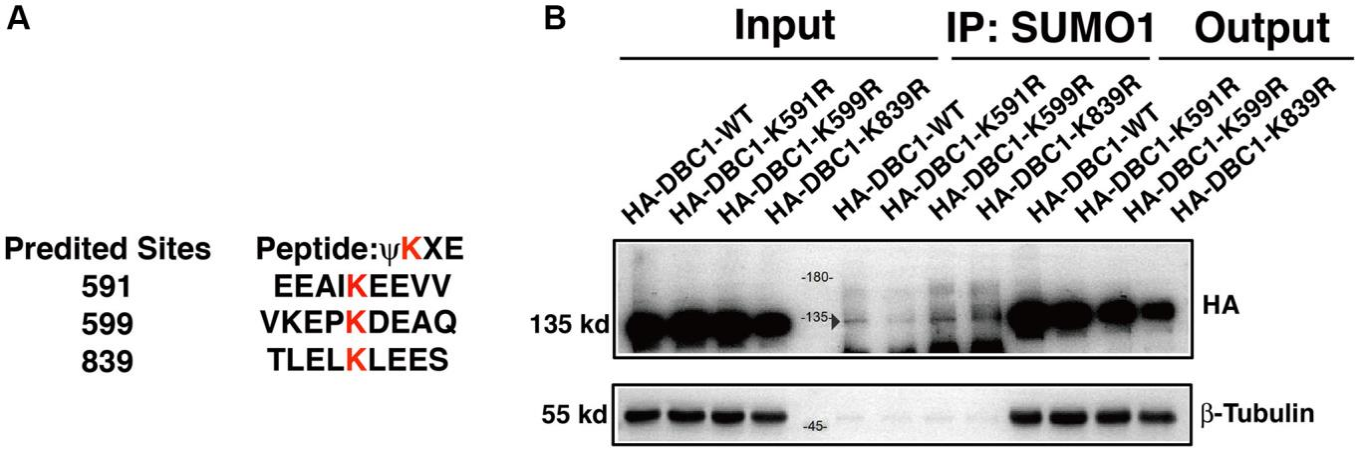
**DBC1 is modified by SUMO1 at K591 in human lens epithelial cells.** (**A**) Three putative lysine residues for SUMOylation of DBC1 were shown in red bold. (**B**) FHL124 cells were transfected with HA-tagged wildtype DBC1 (DBC1-WT) or its K-to-R mutants (K591R, K599R, K839R) as indicated. 24 hours after transfection, cell lysates were immunoprecipitated (IP) with anti-SUMO1 antibody and immunoblottings were performed with anti-HA. β-tubulin was used as a reference for loading.

To identify the SUMOylation site in DBC1 in lens epithelial cells, we used GPS-SUMO software to predict the possible SUMOylation site in DBC1 and identified 3 putative sites: K591, K599 and K839 (Figure 4A). To determine the major residue responsible for SUMOylation of DBC1, we constructed wild-type DBC1 expression plasmid with HA tag, and also generated K to R mutations at the above 3 putative SUMO sites. These plasmids were transfected into FHL124 cells and then harvested for Co-IP analysis. As shown in Figure 4B, SUMO1-conjugation signal at the DBC1 K591R mutant transfected cells were much weaker than that in wild type DBC1-transfected cells, indicating K591R mutation almost abrogated DBC1 SUMOylation. The opposite results were observed in K599R and K839R-transfected cells. Together, these results demonstrated that in lens epithelial cells (LECs), DBC1 is SUMOylated by SUMO1 conjugation at K591 residue.

### Silence of DBC1 attenuates oxidative stress-induced apoptosis of human lens epithelial cells

**Figure 5:**
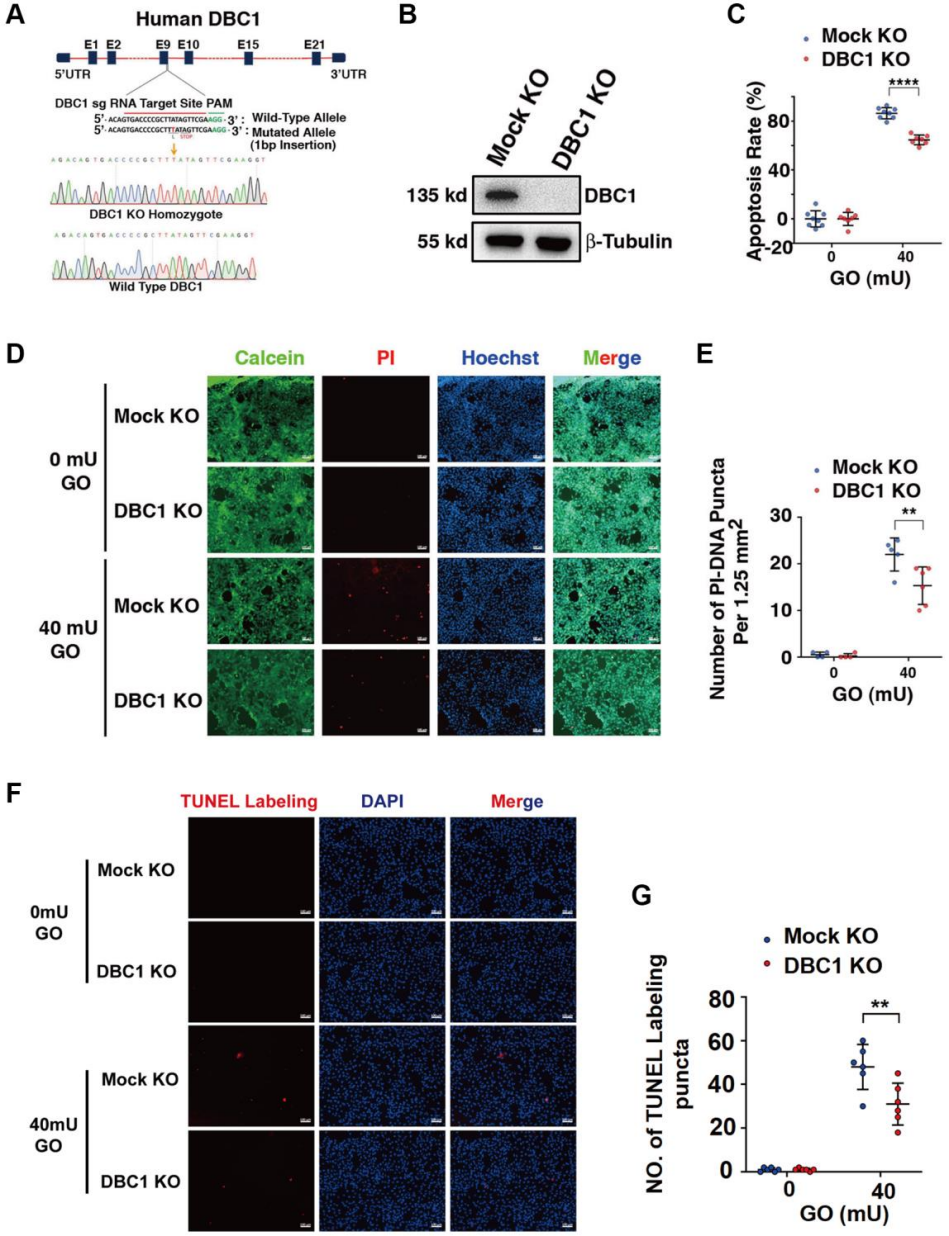
**DBC1 knockout significantly attenuates glucose oxidase (GO)-induced apoptosis.** (**A**) A schematic diagram showing strategy for DBC1 knockout in FHL124 cells by CRISPR/Cas9 gene editing technology. The red underlined base pairs are the sgRNA target, the green underlined base pairs are the protospacer-adjacent motif (PAM). 1-bp base insertion in the mutated allele are shown in red bold. The stop codon introduced in the mutant form is shown. (**B**) Western blot analysis of DBC1 expression levels in control (Mock KO) and DBC1 knockout (DBC1 KO) cells. Note that expression of DBC1 was not detectable in DBC1 knockout cells. The β-tubulin served as the loading control. (**C**) Apoptosis rate changes in Mock KO and DBC1 KO cells under treatment of 40 mU GO for 5 hours were measured by CellTiter-Lumi™ II Luminescent Cell Viability assay analysis. (**D**) Calcein/PI Cell Viability/Cytotoxicity assay analyzed cell apoptosis of Mock KO and DBC1 KO cells under the same treatment as in C. Green fluorescence represents live cells as detected by Calcein-AM, and red fluorescence detected by PI refers to dead cells. Scale bar, 100 μm. (**E**) Quantification of the PI-DNA puncta in panel **D**. ^**^p < 0.01, ^****^p < 0.0001. (**F**) TUNEL labeling assay under the same treatment as in C. Scale bar, 100 μm. (**G**) Quantification of the TUNEL Labeling puncta in panel **F**. ^**^p < 0.01.

To test the function of DBC1 in LECs, we used CRISPR/Cas9 technology to knockout expression of DBC1 in FHL124 cell (Figure 5A). The insertion of a single nucleotide in exon 9 was confirmed with DNA sequencing (Figure 5A) and the absence of DBC1 protein expression was verified by western blot analysis (Figure 5B). Next, we tested if DBC1 deletion could affect the sensitivity of human LECs to oxidative stress-induced apoptosis, we treated the control (mock KO) and DBC1 knockout (DBC1 KO) cells with 40 mU glucose oxidase (GO) for 5 hours, and the cell viability was measured by ATP loss. As shown in Figure 5C, cells with DBC1 deletion showed much stronger resistance to oxidative stress-induced apoptosis than the mock KO cells. Identically, the same treatment was used for live/dead viability/cytotoxicity assay, which revealed that cells with knockout of DBC1 displayed enhanced survival under oxidative stress (Figure 5D, 5E). The apoptotic nature was further confirmed by TUNEL labeling (Figure 5F, 5G). Together, these results demonstrated that silence of DBC1 attenuates oxidative stress-induced apoptosis of human LECs.

### SUMOylation of DBC1 enhances oxidative stress-induced apoptosis of human lens epithelial cells

**Figure 6:**
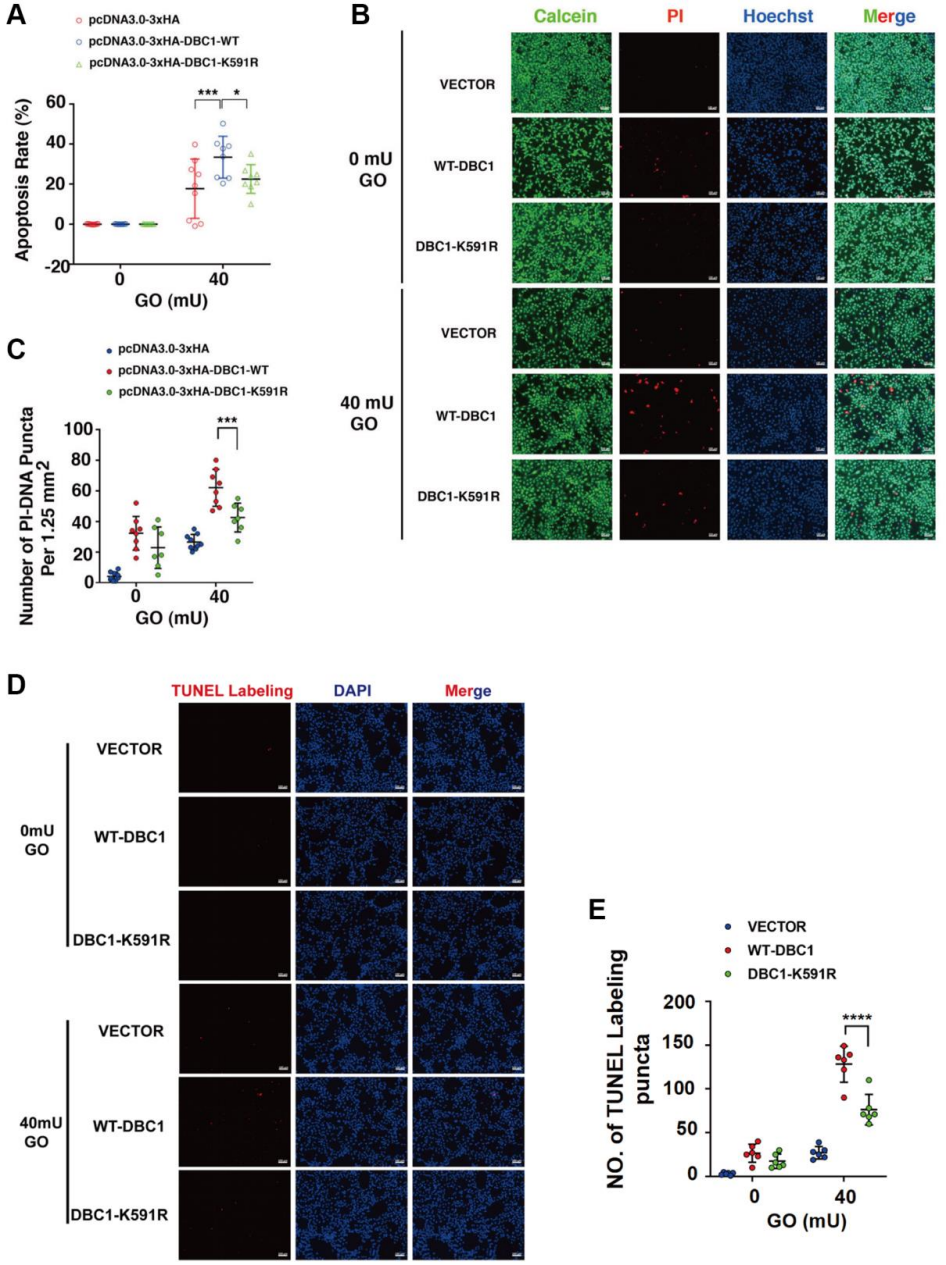
**SUMOylation of DBC1 at K591 enhances oxidative stress-apoptosis.** (**A**) DBC1 KO cells were transiently transfected with HA-vector, HA-DBC1-WT, or HA-DBC1-K591R as indicated. Cell viability assay was conducted to analyze cell apoptosis rate changes in the three types of cells under 40 mU GO treatment for 5 hours. (**B**) Calcein/PI Cell Viability/Cytotoxicity assay analyzed cell apoptosis of the three types of cells under the same treatment as in A. Scale bar, 100 μm. (**C**) Quantification of the PI-DNA puncta in panel **B**. ^*^p < 0.05, ^***^p < 0.001. (**D**) TUNEL labeling assay under the same treatment as indicated. Scale bar, 100 μm. (**E**) Quantification of the TUNEL Labeling puncta in panel (**D**). ^****^p < 0.0001.

To test if DBC1 SUMOylation has an influence on stress response. We transfected the DBC1(−/−) FHL124 cells with HA-vector, HA-DBC1-WT and HA-DBC1-K591R, respectively. 24 hours after transfection, the cells were treated with 40 mU GO and the apoptosis rate was subsequently measured by several methods: ATP loss analysis, live/dead viability/cytotoxicity assay, and TUNEL labeling. As shown in Figure 6A, ATP loss analysis revealed that HA-DBC1-K591R-transfected cells had stronger resistance against GO-induced apoptosis in LECs than HA-DBC1-WT-transfected cells. Live/dead viability/cytotoxicity assay further confirmed that de-SUMOylated DBC1 showed greater resistance to oxidative stress-induced apoptosis than wild type DBC1 (Figure 6B, 6C). HA-DBC1-K591R-transfected cells displayed less apoptotic cells than wild type DBC1-transfected FHL124 cells did (Figure 6D, 6E). Together, these results confirmed that SUMO1-mediated DBC1 SUMOylation sensitizes lens epithelial cells to oxidative stress-induced apoptosis.

### DBC1 interacts with p53 and this interaction is attenuated between DBC1-K591R and p53

**Figure 7:**
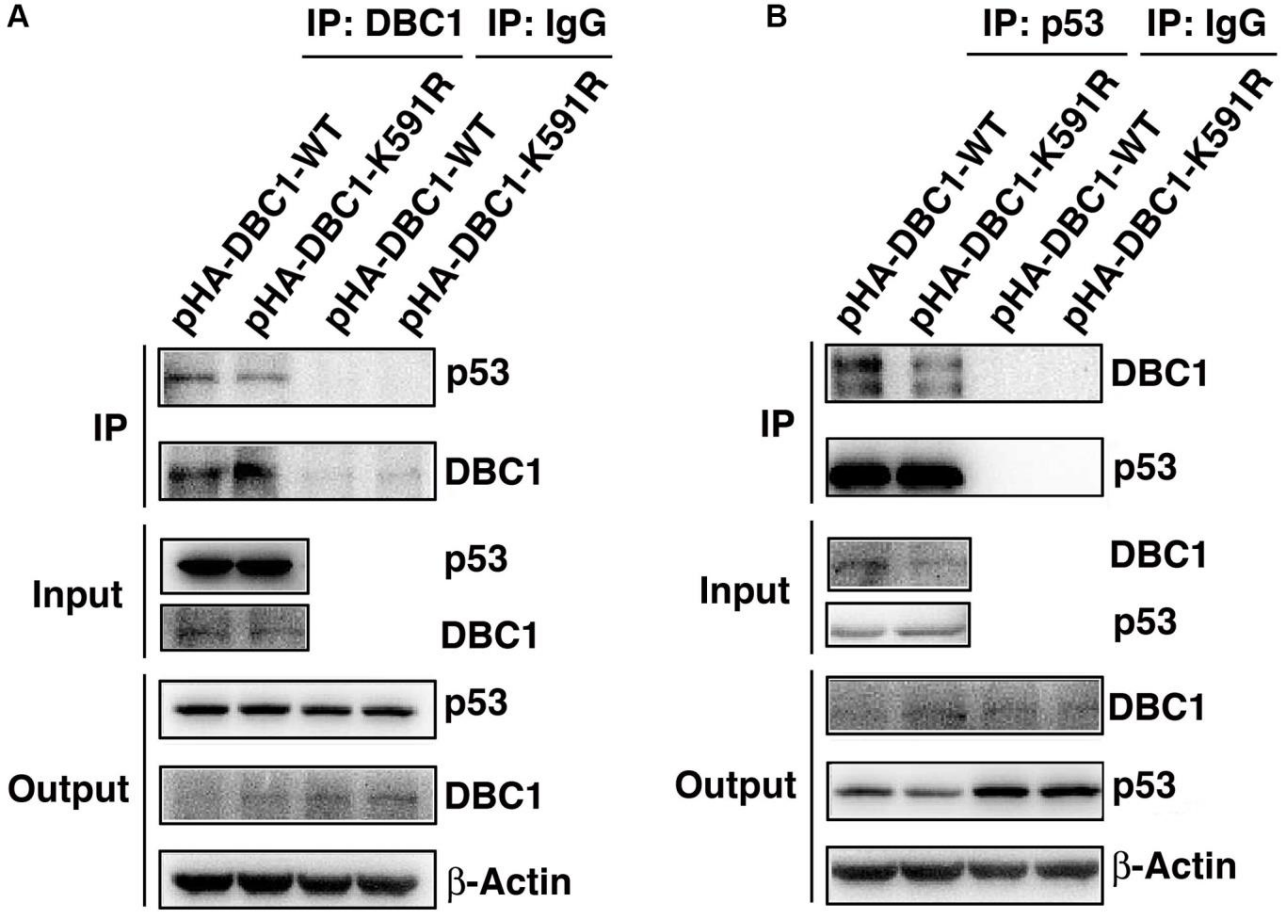
**K591R mutant of DBC1 attenuates the interaction with p53.** DBC1 KO cells were transiently transfected with DBC1-WT and DBC1-K591R mutant. (**A**) Their lysates were subjected to immunoprecipitation with anti-DBC1 antibody followed by immunoblot analysis as indicated. (**B**) Their lysates were subjected to immunoprecipitation with anti-p53 antibody followed by immunoblot analysis as indicated. Note that the K591R mutant of DBC1 weakens its interaction with p53.

Since p53 is a master regulator of apoptosis [32–34], we next explored if DBC1 can regulate apoptosis through p53. We expressed either wild type DBC1 or DBC1-K591R mutant in DBC1 silenced FHL124 cells, and conducted Co-IP assays to compare the interaction between both types of DBC1 with p53. As shown the left panels of Figure 7A, with anti-DBC1 antibody for immunoprecipitation (IP) and anti-p53 antibody for western blot (WB), we detected that the WT-DBC1 displayed clear interaction with p53. The interaction between DBC1-K591R mutant and p53, however, was attenuated to some degree. Similar results were obtained with anti-p53 antibody for IP and anti-DBC1 for WB (Figure 7B). Together, these results confirmed that DBC1 SUMOylation appears to enhance its interaction with p53 in LECs.

### Silence of p53 attenuates DBC1-promoted apoptosis induced by oxidative stress

**Figure 8:**
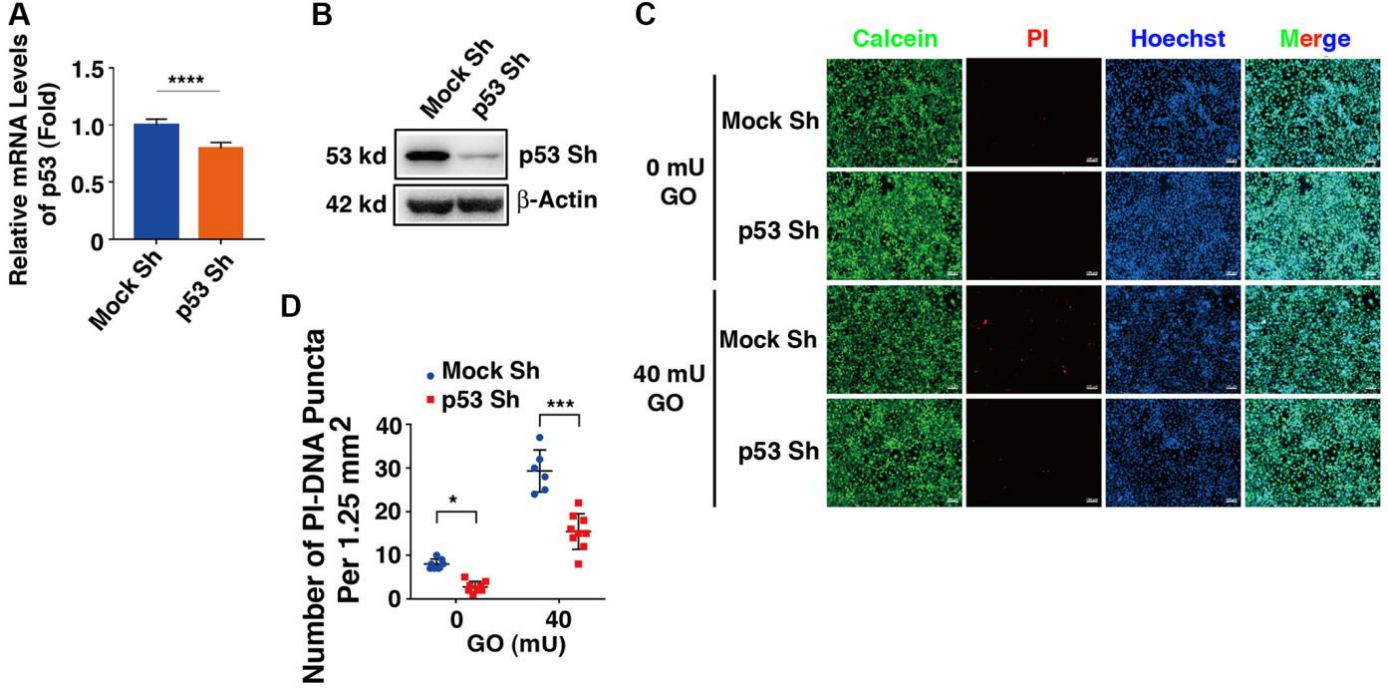
**Knockdown of p53 abrogates DBC1 promotion of oxidative stress-induced apoptosis.** (**A**) qRT-PCR analysis of the mRNA expression level of p53 in FHL124 cells transfected with Mock Sh or p53 Sh. Ct values were normalized by β-actin for each sample. (**B**) Western blot analysis of the protein expression level of p53 in Mock Sh and p53 Sh cells. The β-actin was used as a loading control. (**C**) The Mock Sh and p53 Sh cells were transfected with wildtype DBC1. 24 hours after transfection, the cells were treated with and without 40 mU GO for 5 hours, then Calcein/PI Cell Viability/Cytotoxicity assay was used to analyze cell apoptosis. Scale bar, 100 μm. (**D**) Quantification of the PI-DNA puncta in panel (**C**). ^*^p < 0.05, ^***^p < 0.001, ^****^p < 0.0001.

To test if the proapoptotic function of DBC1 was indeed through p53-dependent pathway, we next established p53 knockdown cells using shRNAs either for nonspecific control (Mock Sh) or targeting p53 knockdown (p53 Sh) through lentivirus infection technology. The p53 knockdown at mRNA and protein expression levels was verified by qRT-PCR and western blot analysis (Figure 8A, 8B). Next, HA-DBC1 expression plasmid was transfected into Mock Sh and p53 Sh cells. After 24 hours, these cells were treated with 40 mU GO for 5 hours. As shown in Figure 8C, 8D, live/dead viability/cytotoxicity assay revealed that lack of p53 led to attenuated levels of apoptotic cells in DBC1-transfected cells induced by GO. Taken together, these results demonstrated that p53 silencing abrogates DBC1 promotion of oxidative stress-induced apoptosis.

### Overexpression of p53 enhances apoptosis in the absence of DBC1 induced by oxidative stress

**Figure 9:**
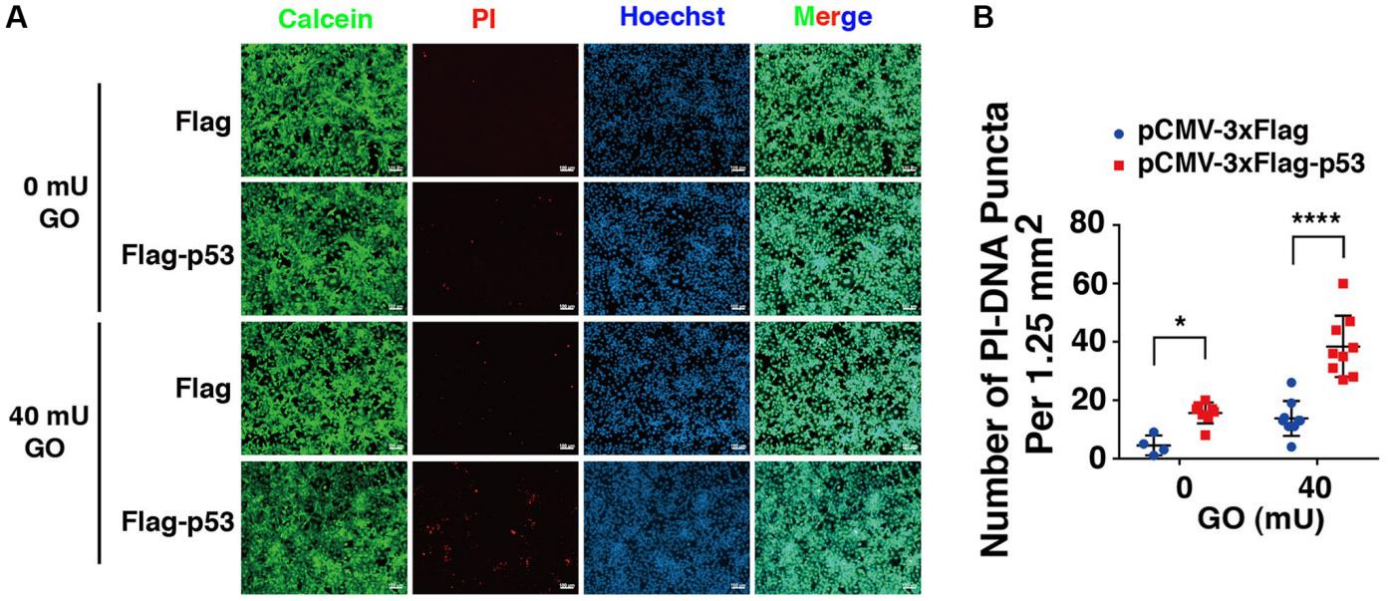
**p53 overexpression overrides the effect of DBC1 absence in promoting oxidative stress-induced apoptosis.** (**A**) DBC1 KO cells were transiently transfected with Flag-vector or Flag-p53 as indicated, then incubating with or without 40 mU GO for 5 hours followed by Calcein/PI Cell Viability/Cytotoxicity assay analysis on cell apoptosis. Scale bar, 100 μm. (**B**) Quantification of the PI-DNA puncta in panel **A**. ^*^p < 0.05, ^****^p < 0.0001.

Next, we overexpressed p53 in DBC1 (−/−) FHL124 cells using Flag-p53 and the Flag-vector as control, and then compared the apoptosis difference. As shown in Figure 9, live/dead viability/cytotoxicity assay revealed that overexpression of exogenous p53 enhanced oxidative stress-induced apoptosis in the absence of DBC1. Thus, overexpression of p53 reversed the impaired susceptibility to oxidative stress-induced apoptosis due to DBC1 loss.

### DBC1 regulates p53 phosphorylation status

**Figure 10:**
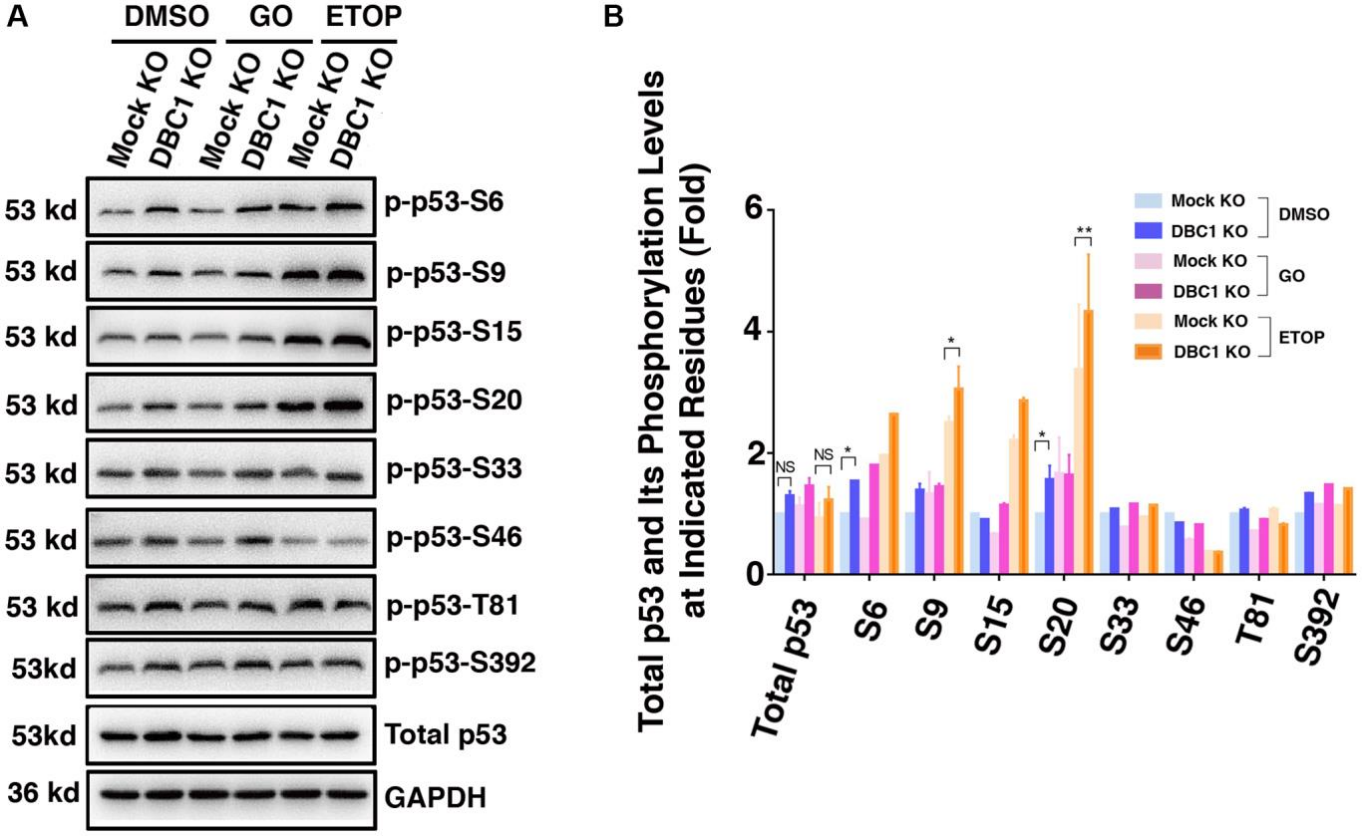
**DBC1 knockdown promotes phosphorylation level of p53.** (**A**) The Mock KO and DBC1 KO cell lines were respectively treated with DMSO, 40 mU GO and 25 μm etoposide (ETOP) for 5 hours, then their lysates were subjected to immunoblot analysis for total p53 and its phosphorylation as indicated. GAPDH served as a loading control. (**B**) Quantification of the Western blot results in panel **A**. All values were expressed as means ± standard deviations. Student *t*-test: ^*^p < 0.05, ^**^p < 0.01 and NS: not significant.

Since phosphorylation of p53 has been implicated in regulating its stability and apoptotic activity [74, 75], we examined whether DBC1 affects phosphorylation status of p53. We used etoposide (ETOP) as a positive control, which has been shown to induce phosphorylation of p53 [76]. As shown in the Figure 10A and 10B, through immunoblot analysis, we detected that knockout of DBC1 significantly promoted the phosphorylation of p53 at S6, S9 and S20. This result demonstrated that DBC1 is capable of modulating the phosphorylation status of p53 and thus regulating its functional status.

## DISCUSSION

**Figure 11:**
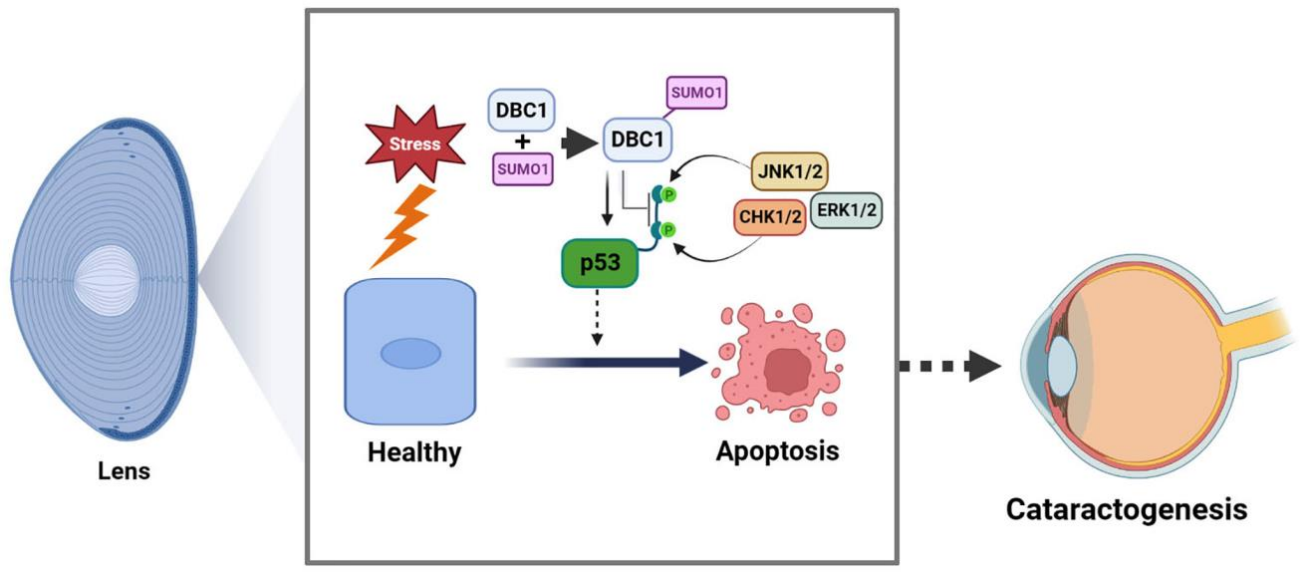
**Model for the role of DBC1 in p53-dependent stress-induced apoptosis.** DBC1 can be SUMOylated at K591 by SUMO1 conjugation in lens epithelial cells. Upon oxidative stress, DBC1 inhibits p53 phosphorylation (derived from upstream kinases such as CHK1/2, JNK1/2 and ERK1/2 depending on the stimuli) to modulate p53 functional status. DBC1 SUMOylation enhances p53-depedent oxidative stress-induced apoptosis, which eventually causes cataractogenesis.

In the present study, we have obtained the followings: (1) DBC1 is highly expressed in human lens epithelial cells and co-localized with SUMO1 in the nucleus; (2) DBC1 can be SUMOylated by SUMO1 conjugation at K591 residue in human and mouse lens epithelial cells; (3) knockout of DBC1 attenuates oxidative stress-induced apoptosis of LECs; (4) DBC1 promotes oxidative stress-induced apoptosis through interaction with p53 to modulate its phosphorylation status at S6, S9 and S20, and its SUMOylation at K591 enhances this interaction with p53. Together, our results identify that DBC1 is an important regulator mediating stress-induced apoptosis. Through promotion of stress-induced apoptosis, DBC1 is involved in control of lens cataractogenesis (Figure 11).

### DBC1 acts as a critical proapoptotic gene to regulate p53-dependent apoptosis in the ocular lens

Apoptosis in the ocular lens plays an essential in both lens development and pathogenesis. During lens development, lens placode invagination and lens vesicle separation with the adjacent corneal epithelium requires apoptosis [6, 77]. Failed undergoing apoptosis of the lens stalk leads to cataract and microphthalmia [78]. Disruption of normal lens development through overexpression of exogenous genes or silence of endogenous genes all causes lens pathology [16–27]. In the adult lens, we and others have shown that induced apoptosis by environment stresses cause non-congenital cataract in both human and different animals [6–15]. Mechanistically, a panel of genes in both Bcl-2 family and caspase family have been shown to play important role [3, 6, 79–83]. Perhaps, the most important regulator is the tumor suppressor, p53 [84–89]. Although developmental apoptosis occurs in both p53-dependent and independent pathways [17, 26], most developmental apoptosis and also the stress-induced apoptosis occur largely through p53-dependent pathway [23, 84–89]. The functional status of p53 can be modulated through phosphorylation by various kinases and p53 binding proteins [74–75]. We have previously demonstrated that the proteins phosphatases PP-1 and PP-2A can modulate p53 function in both lens and non-lens cells [89–90]. More recently, we also showed that Mab21L1 upregulated αB-crystallin can modulate p53 phosphorylation through suppression of the upstream kinases, ATR and CHK1/2 [91]. In the present study, we demonstrated that DBC1, a multi-function gene [39–51], can modulate p53 phosphorylation to regulate its functional status (Figure 10). In contrast to the previous study where it was found that silence of DBC1 attenuates p53 stability [92], here we observed that silence of DBC1 in lens epithelial cells enhances p53 phosphorylation at Ser-6, -9 and-20 (Figure 10). Since p53 phosphorylation at Ser-20 abolishes its interaction with MDM2 [93], our result that DBC1 keeps p53 phosphorylation in check so that both DBC1 and MDM2 can keep p53 level and function in a very fine balance. We also demonstrated that DBC1 can promote stress-induced apoptosis, and thus participate in control of non-congenital cataractogenesis. We demonstrated that DBC1 can promote stress-induced apoptosis which is dependent on p53, and thus participate in control of non-congenital cataractogenesis. Our results are consistent with previous studies where the proapoptotic function of DBC1 was initially demonstrated in 2008 by two independent laboratories [47, 48]. They found that DBC1 negatively regulates deacetylase SIRT1 activity to promote p53-mediated apoptosis. Later it was found that the transducer kinase Chk2 phosphorylates 11S proteasome activator REGγ on Ser247 to increase REGγ-DBC1 binding and p53 acetylation in response to DNA damage [94]. DBC1 can also acts as transcription repressor to regulate apoptosis. During ultraviolet-induced apoptosis, BRCA1 a positive regulator of SIRT1 expression, can bind DBC1 to form a complex in the nucleus and then exported to the cytoplasm to execute its function [95]. In addition, it has been confirmed that the long non-coding RNA MALAT1 interacts with DBC1 to regulate p53 acetylation [96]. Together, DBC1 appears to regulate apoptosis through different targets besides p53. In addition, DBC1 is also associated with immune inflammation. Some studies have shown that DBC1 can inhibit B cell function by negatively regulating NF-κB transcriptional activity [97]. DBC1 also affects the function of regulatory T cells [98]. Whether DBC1 can also regulate these targets in lens epithelial cells are currently under investigation.

### Protein SUMOylation regulates both lens differentiation and pathogenesis

It is well established that SUMOylation is a very important regulatory mechanism, modulating functions of more than 3000 proteins at over 7000 conserved lysine residues [99]. Moreover, SUMOylation is implicated in various human diseases including cardiovascular diseases, cancers and neurodegenerative diseases [100]. In the eye, SUMOylation plays important roles in regulating differentiation of both retina and lens. Pias3-mediated SUMOylation of photoreceptor-specific transcription factors appears to be a key mechanism of rod specification. Normally, the transcription factor Nr2e3 and its upstream regulator Crx function to promote cone-specific gene expression. SUMOylation of these factors converts a cone differentiation promoter into a cone suppressor, promoting differentiation of rod cells in retina [101, 102]. In the ocular lens, we have previously shown that SUMO1-mediated SUMOylation of p32 Pax6 activates its function to regulate early development of both eye and brain [67]. Moreover, SUMO1 promotes lens differentiation and SUMO2/3 inhibits this process. One of the targets modified by SUMOs is the specificity protein 1 (Sp1). SUMO1-mediated Sp1 SUMOylation at Lys-16 positively regulated the expression of lens specific genes coding for β-crystallins, whereas SUMO2/3-mediated Sp1 SUMOylation at Lys-683 prevents expression of these genes [68]. Besides its important roles in regulating lens differentiation, our recent studies demonstrate that SUMOylation is actively involved in lens pathogenesis. First, we have shown that the SUMOylation of total lens proteins is much enhanced in cataract lens than in normal transparent lens [69]. One of such targets is Pax6. Both P32 Pax6 and P46 Pax6 are SUMOylated in cataract patients [69]. Moreover, Pias1-mediated p53 SUMOylation promotes stress-induced apoptosis of lens epithelial cells, thus promoting cataractogenesis [70].

In the present study, we demonstrated that DBC1 is SUMOylated by SUMO1 conjugation both *in vitro* and *in vivo* (Figures 2–4). This is in contrast with earlier studies in cancer cells where DBC1 was found primarily SUMOylated by SUMO2/3 during etoposide-induced DNA damage [103]. Nevertheless, in both cases, K591 was found to be the major SUMOylation site in different tissues. We further showed that DBC1 SUMOylation enhances oxidative stress-induced apoptosis of human lens epithelial cells (Figure 6). Our results are lines with previous studies [104–106]. Since stress-induced apoptosis promotes development of non-congenital cataract [7–15], our results suggest that DBC1 is an important regulator of lens cataractogenesis. Taking together, protein SUMOylation plays important roles in both lens development and pathogenesis.

In summary, our results demonstrated that SUMO1-conjugated DBC1 plays an important role mediating p53-dependent apoptosis and cataractogenesis under oxidative stress (Figure 11).

## MATERIALS AND METHODS

### Animals

The 4-week-old C57BL/6J background mice were used in this study. The mice were raised in a standard barrier facility of Sun Yat-sen University. The room was maintained on a 12-h light/dark cycle and provided free food and water intake. All animal experimental protocols were approved by the IACUC of Zhongshan Ophthalmic Center of Sun Yat-sen University.

### Cell culture

The mouse lens epithelial cell line (αTN4-1) and the human lens epithelial cell line (HLE and FHL124) were cultured in Dulbecco’s Modified Eagle’s Medium (DMEM) (C11995500BT, GIBCO) containing 10% fetal bovine serum (S11150, Atlanta Biologicals) and 1% penicillin/streptomycin (15140-122, GIBCO) as described previously [67–69, 107–111]. The rabbit lens epithelial cell line (N/N1003A) was cultured in DMEM with 10% rabbit serum and 1% penicillin-streptomycin [110]. The ARPE-19 were cultured in DMEM/F12 containing 10% fetal bovine serum and 1% penicillin-streptomycin [71]. All of these cells were kept in a humidified 37°C 5% CO_2_ incubator.

### Plasmids construction and establishment of overexpression cell lines or knockout/knockdown stable cell lines

Human DBC1 cDNA was amplified by RT-PCR from FHL124 cell line mRNA. The cDNA was digested with HindIII and EcoRI and subcloned in frame into cDNA3.0-3×HA vector. The point mutations of DBC1 (K591R, K599R, K839R) were constructed according to the protocol from the QuikChange^®^ Primer Design Program. The p53 cDNA was subcloned into pCMV-3×FLAG at the EcoRI and XbaI restriction sites as described before [70]. To target DBC1 knockout, CRISPR/Cas9 construct was prepared with the oligos annealed and inserted into pSpCas9(BB)-2A-Puro (PX459) vector. The p53 shRNA -1 and -2 were cloned into pKLO.1-TRC vector at EcoRI and AgeI sites. All primers and oligos used are listed in Supplementary Table 1. DBC1 and p53 overexpression cell lines were constructed by transient transfection of FHL124 using Hieff Trans^®^ Liposomal Transfection Reagent from the Yeasen Biotechnology according to the company instruction manual. For DBC1 knockout, PX459-sgDBC1 transfected cells were then subjected to 1.0 μg/ml puromycin selection for 4–6 weeks and subsequently individual clones for the stable cell lines were verified by DNA sequencing and western blot analysis. For p53 knockdown, lentivirus was prepared as previously described [111]. After 1.0 μg/ml puromycin selection, it was verified by qRT-PCR and western blot analysis.

### Glucose oxidase (GO) or etopside (ETOP) treatment

Cells were grown to 90% confluence as described above [70, 111], then replaced with 40 mU GO or 25 μm ETOP prepared by serum-free DMEM for 5 hours. After treatment, all samples were collected for analysis of apoptosis or gene expression.

### Protein extraction and western blot analysis

Total proteins were extracted by RIPA buffer (50 mM Tris-HCl, pH 8.0, 150 mM NaCl, 1% NP-40, 1% sodium deoxycholate, 0.1% SDS, 1 mM EDTA) supplemented with the protease inhibitor cocktail and NEM (SENPs activity inhibitor), and then cell lysates were sonicated and centrifuged at 13,000 rpm for 15 min at 4°C. 40 μg of total proteins in each sample were separated by 10% SDS-polyacrylamide gel and transferred to PVDF membranes. The protein blots were blocked with 5% non-fat milk in TBST (10 mM Tris-HCl, pH 8.0, 150 mM NaCl, and 0.05% Tween-20) and further incubated with primary antibodies overnight at 4°C. The primary antibodies were as follows: SUMO1 (sc-5308, Santa Cruz), SUMO2/3 (11251-1-AP, Proteintech), DBC1 (5857S, Cell Signaling Technology), p53 (2524S, Cell Signaling Technology), p-p53 -S6, -S9, -S15, -S20, -S33, -S46, -T81 and -S392 (9285S, 9288S, 9284S, 9287S, 2526S, 2521S, 2676S, 9281S, Cell Signaling Technology), β-tubulin, β-actin and α-actinin (66240-1-IG, 66009-1-IG, 11313-2-AP, Proteintech). The HRP-conjugated secondary antibody (7077S, 7074S, Cell Signaling Technology) was then applied for 1 h at room temperature. Immunoreactivity was detected with a chemiluminescence detection kit (ECL Ultra; New cell and Molecular Biotech), and the blots were visualized using a Tanon chemiluminescence system (China). The Image J software (National Institutes of Health, USA) was used to measure the intensity of the bands to quantify the protein expression.

### Co-immunoprecipitation

Whole-cell extracts were prepared with lysis buffer (25 mM Tris-HCl, pH 7.4, 150 mM NaCl, 5% glycerol, 1% NonidetP-40, and 1 mM EDTA) and precleared with protein A/G magnetic beads (HY-K0202-5, MCE). Precleared lysates were then incubated with anti-DBC1, SUMO1, SUMO2/3, p53 (the number and brand are the same as above) or anti-HA antibody (3724S, Cell Signaling Technology) overnight at 4°C, followed by incubation with protein A/G magnetic beads for 4 h at 4°C as described before [68, 70–72, 107–111]. The eluted proteins were analyzed by western blots. For detection of SUMOylated DBC1, freshly prepared 20 mM NEM was added during cell lysis.

### qRT-PCR

Total RNAs from cells were isolated using TRIzol reagent (Invitrogen). 1 μg of total RNA was transcribed into cDNA using the HiScript II Q RT SuperMix for qPCR kit (R223-01, Vazyme). Fluorescence real-time quantitative PCR was performed on the LightCycler 480 qPCR system (Roche) with ChamQ SYBR Color qPCR Master Mix (Q411-02, Vazyme) according to the manufacturer’s procedures. The assays were performed in triplicate, and the Ct values were normalized to β-actin. The relevant primers used are listed in Supplementary Table 1.

### Immunofluorescence

Cells were seeded on 24-well glass slides. After PBS wash, cells were fixed with 4% paraformaldehyde for 20 minutes, permeabilized with 0.3% TritonX-100 for 15 minutes, and blocked with normal donkey serum for 1 hour. Then the slides were incubated with the anti-DBC1 (5857S, Cell Signaling Technology.) and anti-SUMO1 antibody (S8070, Sigma) or normal mouse IgG at 4°C overnight. After the PBS washings, the slides were incubated with secondary antibody (4412S, 8890S, Cell Signaling Technology). Cell nuclei were stained with DAPI (D9542, SIGMA) for 5 min. Slides were mounted with anti-fade fluorescent mounting medium (Southern Biotech). Images were captured with a TissueFAXS Q confocal microscope (TissueGnostics, Vienna, Austria). Images were analyzed by TissueFAX Viewer software.

### Apoptosis assays

The cells grown to 90% confluence and treated with 40 mU GO for 5 hours to induce cell apoptosis. The cells were performed with CellTiter-Lumi™ Luminescent Cell Viability Assay Kit (C0065M, Beyotime), Calcein/PI Cell Viability/Cytotoxicity Assay Kit (C2015L, Beyotime) according to the manufacturer’s procedures. The apoptosis rate was obtained by subtracting the ATP value from 1. Images were taken under ZEISS LSM980 Confocal Laser Scanning Microscope. Image J (National Institutes of Health, USA) was used to count PI-DNA positive cells [71–72, 111].

### TUNEL labeling

TUNEL assays were performed using the Vazyme TUNEL BrightRed Apoptosis Detection Kit (A113-03) in accordance with the manufacturer’s instructions. The images were captured with a ZEISS LSM 980 confocal microscope.

### Statistical analysis

Two-tailed Student’s *t*-tests were used for comparisons between two groups. Two-way analysis of variance (ANOVA) was used for comparisons between multiple groups. The error bar in all figures represents means ± standard deviations. The *p*-value < 0.05 was considered statistically significant. ^*^, ^**^, ^***^ and ^****^ represent *p* < 0.05, 0.01, 0.001 and 0.0001, respectively.

### Data availability statement

All data are included here in the article.

## Supplementary Materials

Supplementary Table 1
