# Supplementary material for: SUMO1-regulated DBC1 promotes p53-dependent stress-induced apoptosis of lens epithelial cells
Source: Aging (Albany NY). 2023 Sep 7;15(17):8812–32. doi: 10.18632/aging.205001 (PMC10522365; doi:10.18632/aging.205001)
Supplement: Supplementary Table 1 [file aging-15-205001-s001.pdf]

SUPPLEMENTARY TABLE

Supplementary Table 1. Oligo primers used in the present study.

Purpose	Primer name	Primer direction	Primer sequence (5′-3′)
Plasmids Construction	DBC1-cDNA-WT	F	CCCAAGCTTGATGTCCCAGTTTAAGCGC
		R	GGAATTCTCAGTTGCTAGGTGCCGGCTC
	DBC1-K591R	F	CCAAGGAGGAAGAAGCCATCAGAGAGGAGGTGG
		R	CCACCTCCTCTCTGATGGCTTCTTCCTCCTTGG
	DBC1-K599R	F	GTGGTCAAGGAGCCCAGGGATGAGGCACAG
		R	CTGTGCCTCATCCCTGGGCTCCTTGACCAC
	DBC1-K839R	F	ATCCACACACTGGAGCTGAGGCTGGAGGAG
		R	CTCCTCCAGCCTCAGCTCCAGTGTGTGGAT
Gene Knockout/ Gene Silencing	DBC1-sgRNA-human	F	CACCGTGACCCCGCTTATAGTTCGA
		R	AAACTCGAACTATAAGCGGGGTCAC
	p53-shRNA-1	F	CCGGCGGCGCACAGAGGAAGAGAATCTCGAGATTCTC TTCCTCTGTGCGCCGTTTTTG
		R	AATTCAAAAACGGCGCACAGAGGAAGAGAATCTCGA GATTCTCTTCCTCTGTGCGCCG
	p53-shRNA-2	F	CCGGGAGGGATGTTTGGGAGATGTACTCGAGTACATC TCCCAAACATCCCTCTTTTTG
		R	AATTCAAAAAGAGGGATGTTTGGGAGATGTACTCGAG TACATCTCCCAAACATCCCTC
qRT-PCR	β-actin-human	F	TCACCAACTGGGACGACAT
		R	ATCTGGGTCATCTTCTCGC
	β-actin-mouse	F	TAGGCACCAGGGTGTGATGG
		R	CTCCATGTCGTCCCAGTTGGT
	β-actin-rabbit	F	TGACCAACTGGGACGACA
		R	GTCCTTGCGGATGTCCAC
	DBC1-human	F	GAGGAGTTTGCAGGAGC
		R	GTAGCCACACCAGTTGG
	DBC1-mouse	F	TACAGTTCCAAGGTGCTGC
		R	TGCTTCAGAGGATGCTCTG
	DBC1-rabbit	F	CCACGACTCCAAGAAACGCAAAC
		R	AGTGAGGTGGACCCGGTAGGGA
	P53-human	F	AGTGCTCGCTTAGTGCTCCCT
		R	GTGCGTGTTTGTGCCTGTCCT
